# Systems science methods in public health: what can they contribute to our understanding of and response to the cost-of-living crisis?

**DOI:** 10.1136/jech-2023-220435

**Published:** 2023-06-16

**Authors:** Andreas Höhn, Jonathan Stokes, Roxana Pollack, Jennifer Boyd, Cristina Chueca Del Cerro, Corinna Elsenbroich, Alison Heppenstall, Annika Hjelmskog, Elizabeth Inyang, Daniel Kopasker, Shreya Sonthalia, Rachel M Thomson, Kashif Zia, Srinivasa Vittal Katikireddi, Petra Meier

**Affiliations:** MRC/CSO Social and Public Health Sciences Unit, University of Glasgow, Glasgow, UK

**Keywords:** METHODS, ECONOMICS, HEALTH POLICY, Health inequalities, MODELS, THEORETICAL

## Abstract

**Background:**

Many complex public health evidence gaps cannot be fully resolved using only conventional public health methods. We aim to familiarise public health researchers with selected systems science methods that may contribute to a better understanding of complex phenomena and lead to more impactful interventions. As a case study, we choose the current cost-of-living crisis, which affects disposable income as a key structural determinant of health.

**Methods:**

We first outline the potential role of systems science methods for public health research more generally, then provide an overview of the complexity of the cost-of-living crisis as a specific case study. We propose how four systems science methods (soft systems, microsimulation, agent-based and system dynamics models) could be applied to provide more in-depth understanding. For each method, we illustrate its unique knowledge contributions, and set out one or more options for studies that could help inform policy and practice responses.

**Results:**

Due to its fundamental impact on the determinants of health, while limiting resources for population-level interventions, the cost-of-living crisis presents a complex public health challenge. When confronted with complexity, non-linearity, feedback loops and adaptation processes, systems methods allow a deeper understanding and forecasting of the interactions and spill-over effects common with real-world interventions and policies.

**Conclusions:**

Systems science methods provide a rich methodological toolbox that complements our traditional public health methods. This toolbox may be particularly useful in early stages of the current cost-of-living crisis: for understanding the situation, developing solutions and sandboxing potential responses to improve population health.

**WHAT IS ALREADY KNOWN ON THIS TOPIC**

- Despite considerable interest in systems science methods within public health, there are few practical considerations of how different methods could be applied to contribute insight on a single public health challenge.

**WHAT THIS STUDY ADDS**

- We use the example of the current cost-of-living crisis, felt globally, to illustrate how systems science methods could be used either alone or in combination to shed light on different aspects of this public health challenge.
- We argue that these approaches allow us to better structure complex problems, consider the nature of relationships between key variables, and simulate the impact of plausible policy responses prior to their implementation.

**HOW THIS STUDY MIGHT AFFECT RESEARCH, PRACTICE OR POLICY**

- Systems science methods can provide important insights into the inter-relationships between different drivers of public health problems and highlight how these drivers may be contingent on different contexts.
- These insights are particularly useful in early stages of an unexpected event or situation, complementing other public health methods and making them a valuable source of information for swift policy responses and the targeted allocation of resources.

## Background

Many contemporary public health challenges, such as preventing non-communicable diseases, managing multimorbidity or reducing health inequalities, have proven intractable.^1^ In recent decades, the social determinants of health, the wider set of conditions in which people live and work, have taken centre stage in the framing of public health challenges.^2^ However, progress in addressing these has been slow.^3^ This lack of progress can often be explained by adaptation processes following an intervention, resulting in resistance to change or unintended consequences.^4^ In response, there have been repeated calls for the expansion of systems science methods to better understand today’s public health challenges and thereby intervene more impactfully.^5 6^ However, public health researchers and practitioners often remain unfamiliar with systems science methods, limiting their uptake.^7^

As the WHO outlines,^8^ some of these social determinants are more fundamental for population health than others. For example, those determining socioeconomic position—particularly modifiable factors such as education, occupation and, importantly, income—are often termed structural determinants, while factors such as material circumstances, behaviour and psychosocial factors take a relative downstream position, are often referred to as intermediary determinants.^9^ For a large proportion of other health determinants, such as food and housing, income is an essential factor for access.^2^

From a public health perspective, the current cost-of-living crisis presents a serious challenge.^10^ The crisis, felt globally as prices generally increase across the economy (inflation), is characterised by a fall in real (ie, adjusted for inflation) disposable income since wages fail to keep pace. This means a significant reduction in quantity and quality of goods and services that individuals can purchase.^11^ The potential health and equity implications arising from the cost-of-living crisis are of particular concern. Ultimately, decreased purchasing power affects the health-related decisions any individual can make. This may have implications on the quality of their housing, heating, nutrition, leisure time and interaction with their social networks—aspects affecting both, mental and physical health. Typically, inflation has a stronger effect for more deprived subgroups who need to dedicate a higher proportion of their expenditure to essentials, like food and energy, and are less able to draw on savings to smooth their consumption over rough periods.^12^ Reinforcing this problem, inflation can restrict the support governments or charities are able to provide.

In this paper, we aim to familiarise public health researchers with systems science methods by illustrating how these methods could be used, either alone or in combination, to shed light on different aspects of complex public health challenges. We first outline the potential role of systems science methods for public health research, then provide an overview of the complexity of the cost-of-living crisis as a specific case. We link four selected systems science methods (soft systems methodology (SSMs), microsimulation, agent-based models (ABMs), and system dynamics models (SDMs)) with applications to provide more depth to the relevant case study. We argue that systems science methods provide a unique opportunity to inform proactive policy-making and could help prevent detrimental effects on health, social and economic outcomes.

### Systems science methods for complex public health challenges

Systems science presents a paradigm shift; an acknowledgement that public health challenges emerge within complex systems. A move towards systems thinking has already resulted in a deeper understanding of obesity, substance abuse and smoking, contributing to whole-system intervention design.^13 14^ The WHO has called for systems thinking to now support further efforts to prevent non-communicable diseases.^15^ Yet, the uptake of systems methods in public health research has not kept pace with this development and methods often remain abstract concepts in this research field.^16^

The building blocks of systems are elements: individuals, households, organisations or countries. Elements are interconnected units, but may interact differently over space and time, resulting in a variety of possible outcomes at the micro level, meso level and macro level.^17^ As illustrated in table 1, complex systems can be defined by their properties of emergence, feedback and adaptation, and may exhibit adaptive, self-preserving, dynamic or even evolutionary traits.^18^ Importantly, a complex system is one whose properties cannot be fully explained by the behaviour of its component parts in isolation.^19^ A system can also be part of overarching systems; for example, the healthcare system can be understood as part of a wider system of public services.^20^ These properties of complex systems found in public health often present challenges for traditional epidemiological study designs, which tend to decontextualise the interactions of agents and overlook the potential for feedback and adaptation that occur in real-world settings.^21^

**Table 1:** Glossary summarising the main terminologies within the context of system science

Terminology	Definition
System	A set of elements which are interconnected in various ways that generate identifiable, but not always easily foreseeable, behavioural patterns over time
Complexity	Unpredictable and hard to replicate as a variety of heterogeneous constituent parts behave and interact in various ways, causing changes to existing patterns or completely new patterns of behaviour
Emergence	Describes a property of a system that cannot be applied to the individual constituent parts alone
Feedback	Situation in which a change reinforces, or balances, further change(s) within a system
Adaptation	Agents are capable of learning and adjusting their behaviour to a changing environment. This feature explains the reorganisation of systems in response to shocks and interventions
Tipping points	A threshold after which a system enters a radically new state
Non-linearity	Variables in the system are not related in a simple linear way across the causal chain, for example, through tipping points or feedback loops. Can be different from the statistical interpretation.
Path-dependency	Some processes in systems can be dependent on previous decisions and trajectories

### Cost-of-living crisis as a complex systems problem

The cost-of-living crisis presents a timely case study of a systems problem with public health implications. As a major feature, it affects disposable incomes, a key determinant of health, while limiting the opportunities for efficient population-level interventions.

The systems aspect of the cost-of-living crisis becomes apparent when trying to understand its causes. The International Monetary Fund attributes today’s high rates of inflation to multiple causes. With prices in the economy reflecting the interaction of supply and demand, the current imbalance leading to inflation is mostly thought to be due to constrained supply of goods and services.^22^ These patterns have emerged as part of the interaction and unintended lags of previous policy responses to COVID-19, and its effects have clearly been exacerbated by Russia’s invasion of Ukraine in 2022, and the resulting disruptions in food production and energy supplies.^22^ A country’s ability to adapt and respond to these shocks will also be mediated by their previous broader policy choices, for example, from where they source their energy and goods. In the UK, for example, real income was already falling behind comparator countries, falling 0.2% between 2007 and 2021 compared with an OECD average increase of 0.8%.^23^

**Figure 1:**
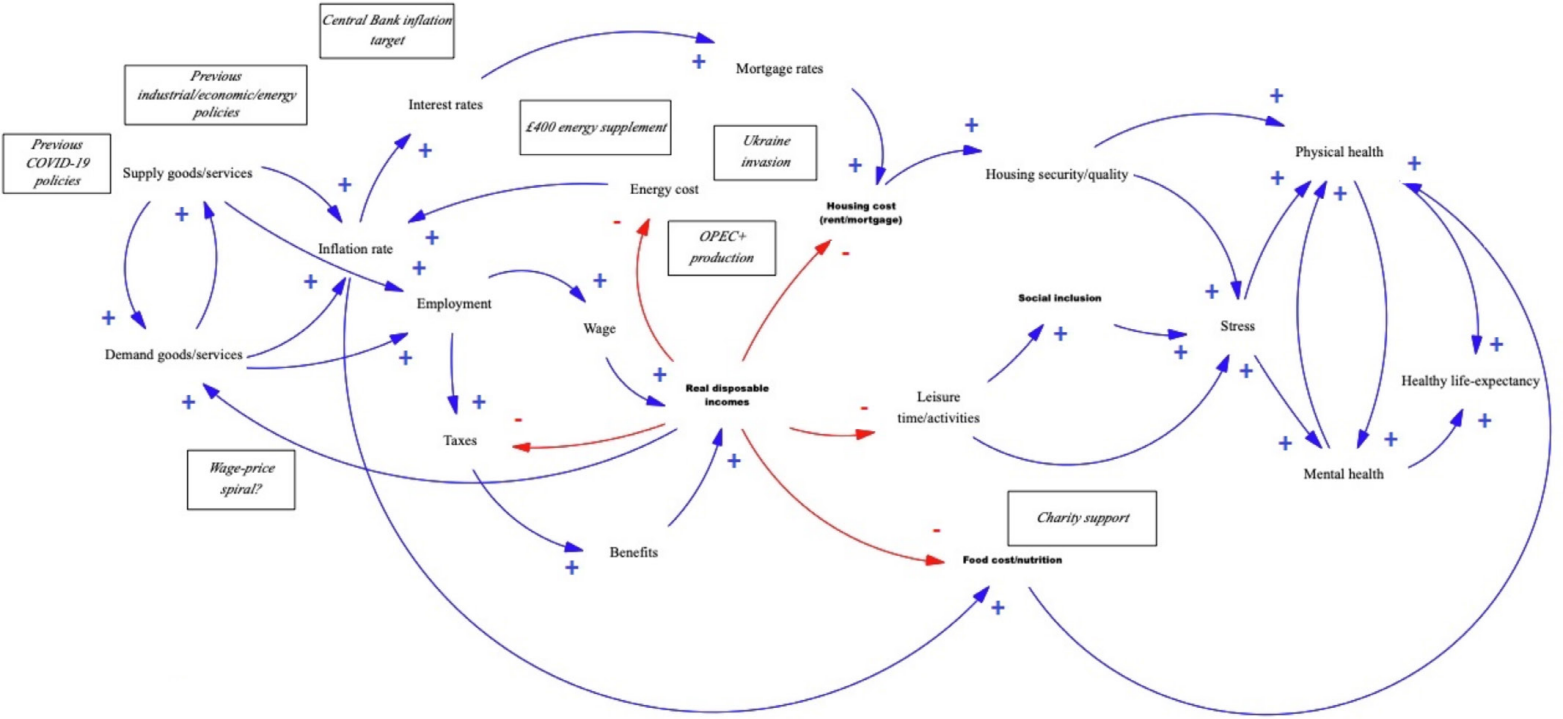


It is often assumed that increasing wages in line with inflation is a straightforward solution. However, disconnecting inflation from wages is often a conscious policy decision to avoid a wage-price spiral, a vicious cycle which might fuel inflation further via increased.^22^ Central Banks try to temper inflation by increasing interest rates, mostly acting to dampen demand rather than the underlying supply problem directly, risking recession and increased unemployment. A simple point of intervention is therefore not straightforward when the wider system is considered, calling for a systems science approach when considering impacts of interventions on population health (see figure 1).^24^

### Systems science methods and cost-of-living crisis

Systems science methods can contribute to informing responses to the cost-of-living crisis. We discuss how four selected key methods (SSMs, microsimulation, ABMs and SDMs) could: (1) help conceptualise the problem and its implications for population health in a policy-relevant way and (2) help produce evidence of simulated system effects and compare trade-offs between potential policy responses from a public health perspective. Previous papers have already dealt in detail with the general introductions to these methods.^6 16^ A summary of each selected method is presented in table 2. An overview of each method’s contribution to public health is given in table 3.

**Table 2:** Brief overview of key systems methods

	Soft systems methodology/ systems mapping	Microsimulation	Agent-based models	System dynamics models
Key purpose	Qualitative approach aimed at providing a better understanding in unclear situations using an action-oriented and iterative approach.	Micro-level simulation model in which changes to a heterogeneous population are modelled via mathematical functions.	Micro-level simulation model with agents interacting with each other, and with and in the environment, given a no of behavioural rules.	Aggregate-level modelling approach examining changes of whole systems over time given the elements, their interconnection, and the systems boundaries.
Tools and building blocks	Rich pictures, root definitions, conceptual models.	Processes informed by existing theory and evidence, combined with real-world data.	Heterogeneous, decision-making agents; behavioural and environmental rules.	Stocks, inflows, outflows, feedback loops.
Data sources	Qualitative; stakeholders and literature.	Predominantly quantitative, micro-level sources of data.	Predominantly quantitative, although expert input and qualitative data can help to frame behavioural and environmental rules.	Predominantly quantitative aggregate-level information; expert domain knowledge, stakeholders and empirical literature.
Method for validation	Must be defensible to stakeholders as models of the defined human activity system.	Internal validation (eg, using subsequent or previous years of the utilised data source) and external validation (eg, similar, but external data source).	Empirical data; sensitivity analysis, pattern-orientated modelling.^38^	Confidence building tests focusing on structure and behaviour; sensitivity analysis.

**Table 3:** Overview of primary focus and contribution to public health research for selected system science methods

Systems science method	Primary focus	Contribution to public health research
Soft systems methodology and systems mapping	Qualitative approach for framing and structuring of problems. Builds systematic understanding of complex, non-linear and ill-defined situations. Includes multiple worldviews across cross-sector actors.	Outlining the boundaries for analysing public health challenges. Integrating different perspectives on public health challenges. Helps to identifies actors’ purposes or preferences to provide common ground for the analysis of public health challenges.
Microsimulation	Processing of microdata representing agents to reflect key processes and relationships within a system. Uses mathematical functions to create long-term simulated projections for units of individuals or households. Perform virtual experiments on complex systems with evolving populations.	Predicting trends in incidence or mortality under alternative health policy scenarios. Estimate population-level effects (eg, costs and benefits) of interventions, policy changes or shifts in health risk factors. Providing ex post and ex ante evaluations of observed and hypothetical policy changes and their impact on public health.
Agent-based models	Processing of microdata representing agents reflecting behavioural rules for agents and key relationships within a system. Uses a range of mechanisms and rules to observe the emergence of the macro-level phenomena for individuals or households’ interactions. Perform virtual experiments on complex systems with evolving populations.	Particularly useful when real world experiments are not feasible or possible, for example, due to the scale and character of the studied intervention. Enables moving from an abstract representation of a simplified system to a simulation of a well-defined, heterogeneous population. Allows to examine the role of micro-level decision-making and interactions at the micro level for population health outcomes.
System dynamics models	Modelling aggregate-level outcomes emerging in systems and subsystems as a result of inflows, outflows and feedback loops. Iterative process of continued systems mapping and simulation until model mimics systems behaviour of interest. Aid intersectoral engagement in policy processes.	Understanding the role of specific elements within systems and sub systems for public health outcomes. Evaluation how shocks and policy responses are likely to impact public health outcomes. Identification of unintended consequences at the aggregate level, including the public health effects of policy responses.

### Soft systems methodology and systems mapping

SSM is a qualitative method for a holistic framing of systems.^25^ It is an action-oriented process of inquiry and is frequently conducted through workshops and interviews. SSM offers a clearly defined cycle to guide users through a process of both learning, and identifying actions to be taken to improve complex, unclear (social) situations. This cycle involves identifying the problematic situation; initial drawing of Rich Pictures to illustrate the situation and the relevant elements and relationships present within it; translation of these pictures to conceptual models representing different worldviews of the variety of stakeholders involved, and possible pathways for addressing the problem; comparison of the conceptual models with the real world to gauge discrepancy; feasible and acceptable actions for improvement considered and enacted to address the problematic situation.^26^

Setting the system boundaries of the problem to examine can be a challenge for each of the following systems science methods discussed. Which elements of the system to focus on can quickly become unwieldy. This is particularly true for a problem like the cost-of-living crisis, which cuts across many domains and potentially affects most, or all, population subgroups in different ways. SSM can help reign in the problem, to prioritise which aspects are important for different stakeholders. For example, we might want to focus on the impact decreasing household incomes and government spending might have on child health outcomes. SSM could help identify and bring together relevant stakeholders to understand priority risks and possible (or: feasible/acceptable) interventions to address these risks. For example, this could result in a more focused and defined causal loop diagram, mapping out what problems and solutions each policy sector is working on and how things could be connected to attempt to alleviate the crisis and address specific child health outcomes. These causal loops could also subsequently be combined with other methods for quantitative simulation, outlined below.

### Microsimulation

The second complexity method relevant to our case study is microsimulation. Microsimulation models can provide a virtual simplification of the real world. They use disaggregated data, often individual or household-level data, and are therefore well suited to studying impacts on health inequalities. Informed by the integration of existing theory and evidence, they combine real-world data with mathematical functions to create simulated projections for highly varied (heterogeneous) individual units. The processes simulated are a set of transition probabilities for movements between states, for example, from a *healthy* to a *disease* state.^27^ The models can be classified as either static or dynamic. Static models provide arithmetic predictions of morning-after-effects from a change in the system, such as a tax or benefit policy.^28 29^ Dynamic models, instead, provide a synthetic world to perform virtual experiments on an evolving population.^27^

One of the immediate concerns for the public health community might be the expected health impacts of the cost-of-living crisis, to prepare and equip future community and health system resources and prioritise future research. Microsimulation is an established method that can help inform this and allow comparison under different policy scenarios. For example, mental health outcomes might be a particular concern in the shorter term with imminent financial stress and poverty. The Heath Equity and its Economic Determinants model combines output from EUROMOD—a static microsimulation model used by the European Commission to inform decisions on the distributional impact of changes in tax and benefit policies^28^—with a dynamic microsimulation model to project the distributional effects of these policies on mental (and general) health outcomes over multiple years.^30^ A simplified version of this approach has already been used to illustrate the implications of the cost-of-living crisis, showing outcomes under different energy price cap policies for the UK.^31^ For a better understanding of the cost-of-living crisis, a dynamic model capturing the change in value of income in different periods could be used to model the reduction in household spending on healthcare or health benefiting activities. This could provide long-run projections for possible health effects of the crisis for public health preparation and budgeting.

### Agent-based models

ABMs are computational models where agents, the unit of analysis in the system, interact with each other and their environment. These dynamic and adaptive interactions separate them from microsimulations, adding to the complexity they can model.^32^ The rules of engagement and behaviour of agents can be determined from simple heuristics or defined based on theory or data. Environments situate agents in a representation of space, which can either reflect actual physical space (eg, geographical information systems modelling of real cities^33^), or completely simulated space. As agents interact, they adapt their behaviour which has system-level consequences.^34^

ABMs are particularly strong for understanding how knock-on, or unforeseen, system consequences can result from changes at the individual level. They provide a possible and plausible explanation for an emergent macro-level outcome, showing how this can emerge, given a set of assumptions and constraints. For example, devalued income is likely to have impacts on spending and purchasing behaviour of individuals, which might differ substantially by population subgroup. ABMs could be used to explore how people might subsequently change their spending on unhealthy commodities (eg, alcohol, tobacco, gambling) and how these spending decisions may differ between groups depending on social influences (eg, norms) and what is available in their local environment (eg, off-licences and betting shops). ABMs could also allow examination of the mechanisms of changes in spending impacting on employment in different sectors, such as retail, and the resulting population health consequences. For example, cuts and restrictions of spending on various services (eg, cleaners, gardeners, dog walkers) and potential impacts on the affected individuals’ health through increased stress, precarity of employment and further reduced income. Examining how system effects might change with different policy scenarios is again possible by varying the environment the individuals might face, or the combination of individual circumstances that might be experienced under different public health policy interventions.

### System dynamics models

SDMs enable modelling systems at an aggregate level, making them an ideal choice for testing policies or wider implications of shocks.^5^ SDMs are built on the idea that stock changes over time can be modelled by their interdependence on in- and out-flows, feedback loops and lag effects. One key feature of SDMs is the strong stakeholder engagement nature which lends itself to early collaboration. Like SSM, the model building process itself can aid shared understanding of the complex problem and involve relevant intersectoral stakeholders in the policy process. Like microsimulations and ABMs, SDMs allow ex ante forecasting of a systems’ behaviour and estimation of alternative outcomes given different potential public health policy choices. The simulated long time horizons are a key strength of SDMs, particularly since public health deals with upstream causes of the causes, where it is extremely difficult to directly capture lagged health effects causally with empirical research.^35^

Since the cost-of-living crisis is ultimately a whole-economy problem, many of the population health solutions might necessarily be explored at this aggregate level. For example, rising energy prices have been identified as a key driver of recent acute escalations in prices, particularly in Europe and the UK. Energy prices are a key input for basically every other sector, including food production and transport, and supply of any other product or service that affects population health. Unfortunately, energy also represents an input where demand, a major focus of Central Banks, is difficult to reduce without causing many knock-on consequences across sectors, for instance, unemployment with decreased production and sales. Modelling the aggregate interactions and these key drivers of current inflation might help to explore ultimately more effective public health solutions in the long term. For example, it could allow modelling of windfall taxes and other direct energy policy options, and comparison of the relative benefits of up/downstream policy efforts on stock of population health as a key outcome of interest. SDMs could also model the impacts on specific subsystems, or impacts on other social determinants of health, such as the housing market, the provision of third sector services, or health and social care capacity.

Overall, this paper has covered four of the main systems science methods and discussed how they can be applied to the cost-of-living crisis. Table 4 provides an overview of strengths and limitations of the selected methods. Importantly, these methods can be combined in hybrid-models to attempt to further dampen these limitations.

**Table 4:** Strength and limitations of system science methods for application in public health research

Systems science method	Strengths	Limitations
Soft systems methodology/systems mapping	Framing complex problems as clearly defined systems accounting for multiple and conflicting perspectives. Practical framework to prioritise feasibility depending on resource allocation and variation in stakeholders' purposes and perspectives.	Insufficient accommodation of differing views or a dominating perspective can lead to inaction of stakeholders. No direct guidelines on the implementation or management of the iterative actions.
Microsimulation	Focus on heterogeneous populations enables analysis of distributional effects of changes. Aggregated outcomes provide long-run population-level projections for different policy scenarios. Provide immediate evidence for long-term effects from multiple factors interacting within a complex system.	No explicit interactions between simulated units in the model. Typically require attribute-rich and substantial quantitative data.
Agent-based models	Bottom-up, individual-level perspective, incorporating heterogenous agents. Agents can be explicitly spatial. Modelling of macro-level outcomes based on interactions at the individual level which may change over time.	Time-consuming and difficult to validate, lack of real-world data. Often substantial assumptions required to assign attributes to agents and define behavioural rules. High computational demand for more complex models.
System dynamics models	Forecasting of an entire systems’ behaviour and estimation of alternative outcomes given different potential policies ex ante. Simulated long time horizons to account for upstream causes of the causes of public health issues, where it is extremely difficult to directly capture lagged health effects causally. Strong intersectoral stakeholder engagement aiding early collaboration and informing urgent policy processes.	Requires substantial assumptions with respect to causal relationships. Often lack of evidence-base for lag times across causal chains. Interpretations of forecasts are conditional imprecise.projections rather than accurate point estimates. Relative inability to explicitly model heterogeneity or inequalities themselves, for example, between population subgroups.

## Discussion

Systems science provides a rich suite of methods for approaching today’s public health challenges, such as the current cost-of-living crisis. All four methods presented in this paper can substantially contribute to address public health concerns. We acknowledge that our overview is not exhaustive, and that other methods could potentially meet these goals, but we address the relevant systems science methods that meet the paper’s aims.

All presented methods complement existing methods, more commonly used in epidemiology and public health, and together could provide better evidence for policy. While existing epidemiology and public health methods excel at defining an overall problem and evaluating policy solutions after implementation, systems science methods help fill key gaps in early policy development stages.^15^ Their ability to simulate possible implications and interactions prior to implementation can allow sandboxing of plausible effects before costs are sunk. Similarly, applying this complexity lens offers a shift in the conceptualisation of public health as systems of interconnected and interdependent parts and focusing on the mechanisms relevant for explaining a given phenomenon and for intervening in a transparent way.

All presented methods take existing public health literature and knowledge as key inputs, for example, when building models or informing relationships within the system. Many allow the amalgamation of quantitative and qualitative data, which are often quite separately considered—even in mixed-methods studies. With early engagement, they might also allow more useful roll-out of policy and data collection for subsequent rigorous causal policy evaluation, using methods such as natural experiments which are already strongly used in this field.^36^ This is especially important, because any simulation is necessarily a simplification of the real world and based on assumptions, requiring empirical testing and refining to improve models for their next use iteratively.

Our call for a stronger systems science focus echoes previously raised opinions within the public health research community.^1 6 7 15 16 21 37^ We argue that a stronger understanding of systems science methods and their different approaches to conceptualising research questions, and especially their potential range of applications, is required to match this unmet need. Systems science methods should not be seen in opposition to more traditional methods. Instead, synergies can be realised when both are used in symbiotic and complementary ways.^16^

Considering the health and equity implications of today’s public health challenges, ignoring complexity cannot be an option. Systems science methods can help make complex realities explicit, particularly in a crisis when solutions are urgently required.

## Data Availability

No data are available. Not applicable to this review article.
